# Multi-Level Barriers to Prison Mental Health and Physical Health Care for Individuals With Mental Illnesses

**DOI:** 10.3389/fpsyt.2022.777124

**Published:** 2022-06-03

**Authors:** Kelli Canada, Stacey Barrenger, Casey Bohrman, Anthony Banks, Punita Peketi

**Affiliations:** ^1^School of Social Work, University of Missouri, Columbia, MO, United States; ^2^Psychiatry, Northeast Ohio Medical University, Rootstown Township, OH, United States; ^3^School of Social Work, West Chester University, West Chester, PA, United States

**Keywords:** prison health care, mental illness, qualitative methods, healthcare experience, interactions with correctional staff

## Abstract

**Objectives:**

People with mental illnesses are overrepresented in the U.S. prison population. It is well established that incarceration for this population poses physical and mental health risks including greater likelihood of victimization and suicide compared to the general prison population. Yet, research is less clear about how staff and services shape these prison experiences. The aim of this study was to examine how people with mental illnesses experience incarceration through interactions with correctional officers and treatment staff and their use of physical and mental health care services.

**Methods:**

This project utilized a non-experimental design and qualitative research approach to address the research aims. Adults with mental illnesses who were formerly incarcerated were recruited from three different sites in the Midwest and East Coast. Participants completed an in-depth interview and brief survey on health histories. Data were analyzed using descriptive statistics and the framework method for qualitative analysis.

**Results:**

Participants (*n* = 43) identified challenges to utilizing health and mental health care including perceived access and quality of mental health, medical, or substance use treatments obtained during prison as well as participant's willingness to engage in services. Access to health care was marked by cumbersome procedures required for service use requests and inadequate staffing. Participants reported mixed experiences with medical and mental health staff ranging from experiencing kindness to feeling staff did not believe them. Participants perceived most correctional officers as exhibiting professionalism while some enacted stigma and created additional stressors.

**Conclusion:**

Interactions with correctional staff and health care services have the potential to buffer the stressors and risks inherent in prisons for people with mental illnesses. Perceptions from participants suggest both individual- and systems-level opportunities for intervention to better support people with mental illnesses in prison.

## Introduction

Mass incarceration disproportionately affects people with mental illnesses. The prevalence of mental illnesses among people incarcerated varies from a low of 2% to a high of 48% across studies; for most disorders, this rate is higher than estimated rates in the community (i.e., for any mental illness, approximately 20% of U.S. adults) (1–3). The prison environment is risky for all people. Living in prison poses numerous health and mental health risks including loss of autonomy, self-worth, and self-esteem (4), high mortality during and after a person's prison sentence (5, 6), higher risk and exacerbation of chronic diseases and comorbid medical conditions (7, 8) and exacerbation of psychiatric symptoms (9, 10). For people with mental illnesses, in particular, prison poses high risk for physical and emotional trauma that acts as an acute and chronic stressor throughout incarceration (4). Specifically, people with mental illnesses are at heightened risk of physical and sexual victimization (11, 12) and suicide (12–14). Once involved in the criminal-legal system, people with mental illnesses face elevated risk of re-incarceration due to parole and probation violations and new arrests (15).

Prisons were not designed to be clinical treatment facilities and they are not funded sufficiently to offer the comprehensive care that people with serious mental illnesses require, yet they are some of the largest providers of mental and physical health services in the United States. Unmet physical and mental health needs in prison impacts people during incarceration and re-entry back into the community (5, 16). Thus, accessing quality services during incarceration is essential to treating existing and emergent conditions and reducing the health risks that people face during re-entry into the community. There is limited research, however, on using prison-based healthcare services from the perspective of people who were formerly incarcerated. In order to address this limitation, this current project examined the experiences of people with mental illnesses in accessing and using health and mental health services during incarceration.

### Prison Health Care: An Overview

Olson et al. (17) argue that prison healthcare standards are “piecemeal and poorly defined” (p. 1). In fact, accreditation for prison health care is voluntary and not regulated in the same way hospitals and clinics are regulated in the community. This can produce variation in service delivery and quality of care as well as a lack of oversight on the use of best practices in medical and mental health, leading to potential ethical violations. Despite legislation requiring people in custody to receive adequate health care [see Estelle v. Gamble, (18)], established standards for what constitutes adequate care is largely driven by ongoing litigation rather than the promotion of correctional best practices (19). One example of the wide variation in health care practices is how much state prisons spend on healthcare for people incarcerated. In fiscal year 2015, California spent an average of $19,796 per person while Louisiana spent an average of $2,173 per person (19) .

Prisons vary in their service delivery models as state prisons may employ healthcare workers as state employees, contract or outsource to a third-party, or use a hybrid model of care involving a mix of state employees and contractors (19). Prisons often have a reception center that is centrally located in each state. Individuals receive risk assessments and health screenings at the reception centers, which determines the prison individuals will be assigned. Once individuals arrive at their longer-term prison, they may receive a more thorough health assessment particularly if they have a chronic health or mental health condition. Once individuals are assigned to units within the prison, acute health needs may be requested, as needed, through an established process. While exact procedures vary from institution to institution, they follow the same basic principles: individuals make a request by filling out a medical slip which is deposited into internal mail or collected by correctional officers who then transport slips to the appropriate medical clinic. Individuals are then informed of the date of their clinic appointment. Like community clinics, chronic health conditions are addressed by following the established plan of care which may include medications, monitoring, or therapy.

Although research on the use of health and mental health care in prisons is limited, existing research finds no difference in health care use within prisons across men and women. Incarcerated women, however, do have higher levels of disease burden (i.e., higher prevalence of health conditions) yet less use of prison health care resources (20). Across all racial groups, Black men are most likely to utilize heath care services in prison (20). Older adults, however, typically have higher healthcare needs yet face more barriers and obstacles to using health services in prison including distrust in services, perceiving negative consequences due to help-seeking, and environmental obstacles (e.g., infrastructure) (21). Additional research is needed to better understand patterns of healthcare utilization in prisons across different types of prisons (e.g., vary security levels) and subpopulations (e.g., people with mental illnesses).

### Prison and People With Mental Illnesses

Although initiatives like jail diversion programs and mental health courts are intended to keep people with mental illnesses in the community, they remain overrepresented within the prison population (22). The high prevalence rates of mental illnesses among people in prison is due, in part, to people with mental illnesses spending an average of 15 months longer in prison than people without mental illnesses, even when charged with similar crimes (23). They are also more likely to serve their entire sentence rather than qualify for early release or parole (9). Individual differences in the ability to adapt to prison, limited healthcare and programs within prisons, social isolation, segregation, and stress resulting from risk of violence and prison conditions can lead to adverse health and mental health outcomes (24, 25). Living in prisons has negative impacts on health and mental health, which effects people while in prison and after they return to the community. However, improvements in mental and physical health while in prison and post-release can drastically reduce the likelihood of violations during prison and re-engagement with the criminal justice system (26, 27).

Although the prison environment is risky for people with mental illnesses, there is limited understanding of how staff action or inaction and the use of clinical treatment and services impact physical and mental health outcomes. It is also unclear how these services, treatment, and supports may buffer or contribute to the negative impact of prison. Research does support the key role that prison staff play in facilitating access to rehabilitation services and treatment (28). However, it is unclear how people with mental illnesses experience their interactions with staff (e.g., supportive, coercive) and whether interactions result in quality care. Watson and Meulen (29) stress the importance of qualitative research to address this gap in prison research. Given that diversion programs are only reaching a fraction of people with mental illnesses, it is critical to develop knowledge about prison health and mental health care and individuals' experiences with staff to reduce the short- and long-term negative impacts of prison. To better understand prison health and mental health care, this study explored the experiences of formerly incarcerated adults with serious mental illnesses regarding their interactions with staff and their experiences using health, mental health, and substance use treatments while incarcerated. Patient-centered research is largely absent in corrections-based work, particularly among studies involving people with serious mental illnesses. The perspectives captured in this project contribute to the current body of literature as the perspectives of people with lived experiences are key in making changes within prison healthcare systems.

## Materials and Methods

The aim of this study was to examine how people with mental illnesses experience incarceration by focusing on interactions with correctional officers and treatment staff and use of physical and mental health care services. We utilized an exploratory, non-experimental design and a qualitative approach to address the research aims. This is the best approach to understand the complexity of a topic or issue (30). For this project, 43 adults with mental illnesses who were formerly incarcerated were recruited from three different sites in the Midwest and East Coast. Participants completed an in-depth interview at one time point and short surveys on their health histories. This project was approved by University Institutional Review Boards at the three recruitment sites. Data were merged after the data collection ended and all identifying information was removed. Participants were required to provide consent for study participation and audio recording.

### Sampling and Recruitment

Formerly incarcerated people with mental illnesses can be a hidden population and difficult to access. As such, a tiered sampling approach was utilized, beginning with purposive sampling from community mental health settings. Purposive sampling occurs when the researcher selects cases strategically to provide depth into the phenomenon under study; the cases selected are meant to include study participants who are most able to engage in a dialogue regarding their experiences in prison to shed light on the concepts under study (31). Snowball sampling was the second sampling approach as participants were also invited to hand out flyers about the study or provide study information to people in their networks. In order to access the sample, researchers initially posted flyers in community mental health setting in the three respective communities. The flyers instructed interested participants to call the researchers or speak with their treatment provider about their interest. Interested participants either called researchers directly or asked one of their providers for assistance with calling researchers. After a participant completed the interview, they were also provided with several flyers and were invited to hand them out to people in their network. These flyers were the same as the ones posted in agencies. The aim was to recruit 15 participants per geographical location, or until topical saturation at each site was reached.

Eligible participants were English speaking adults (18+) and diagnosed with at least one serious mental illness (i.e., major depressive disorder, any schizophrenia-spectrum disorders, bipolar I or II). Eligible participants also had a history of incarceration in a state, medium- or maximum-security prison within the past 3 years. Screening took place over the phone. All participants screened met eligibility criteria and were able to provide informed consent. No participants refused to participate.

### Procedures and Measurement

Participants completed a 2-h, face-to-face meeting that involved an in-depth interview and a brief questionnaire. The in-depth interviews consisted of a series of questions to prompt participants and began with a broad question, “*Can you please tell me about your experience in prison?*” This allowed participants full discretion in how they described their experience and reduced the risk of interviewers biasing participant reporting. Interviewers engaged conversationally with participants while ensuring they followed the guide of questions. Interviews covered the following: experience interacting with correctional officers and other prison staff; health and mental health during prison; and use of treatment and support services in prison. Interviews were audio-recorded and transcribed by a third-party. Researchers developed a brief questionnaire to gather information on demographics, current living situation, medical insurance, use of entitlements, major diagnoses (i.e., mental, physical, and substance use), drug use, lifetime arrests and arrest history, jail and prison admissions, and length of stay in detention to supplement in-depth interviews. Interviews were conducted by PhD-level researchers at two sites. A PhD-level researcher and one Master's-level social work student completed interviews at the third site. The PhD-level researcher at the third site trained the student and was present during all interviews to assist the student, as needed.

### Data Analysis

Multiple approaches to data analysis were used in this study. In-depth interviews were analyzed using the framework method (32). The framework method is a systematic and iterative approach to analyzing qualitative data in teams for research that aims to describe and explain a phenomenon. It is within the family of thematic analysis and includes several structured steps to carry out the analysis. For this project, the specific steps used are detailed. First, the audio files were transcribed and reviewed for accuracy. Researchers read through the transcripts to become familiar with them and created notes and memos to record impressions of the data. Codes were developed inductively through transcript reviews; two of the authors reviewed two transcripts each and drafted a codebook with definitions of codes and examples. Four researchers (two Ph-D prepared, one doctoral student in social work, and one medical student) completed line-by-line coding of three transcripts. Following this coding exercise, the team met and discussed the meanings of codes and any gaps in the current codebook. Once the codebook was finalized, the four researchers each independently coded all transcripts, meeting regularly to discuss the coding process and any emergent themes in the data. The codebook included 21 parent codes; 12 of the parent codes included several child codes, as well. Any coding discrepancies were discussed in team meetings using a consensus approach. These discussions allowed for iterative enhancements to the codebook to increase rigor in the process of coding. Data analysis was organized using Dedoose, Version 8.3.43 and 9.0.17.

The brief questionnaire was analyzed using descriptive statistics including frequencies and measures of central tendency. Six participants from one of the geographic locations did not complete questionnaires; these six participants are not reflected in the demographic information listed below. Calculated percentages in the results are based on the 37 participants who did complete the questionnaire. Minimal data were missing from the other participants. If data were missing, the case was removed from the analysis of the missing variable but was not dropped from the dataset completely.

## Results

A total of 43 participants took part in this research. They ranged in age from 27 to 62 with an average age of 45.6 (*SD* = 9.3). The majority of the sample identified as a man (*n* = 34, 91.9%). Participants self-reported their racial and ethnic identities as Black or African American (*n* = 14, 37.8%), White (*n* = 15, 40.5%), Biracial (*n* = 4, 10.8%), and Latinx (*n* = 4, 10.8%)^1^. Many participants had a high school diploma (*n* = 20, 54.1%) or did not attend school past middle school (*n* = 7, 18.9%). Ten participants were currently on disability for their psychiatric illness (27.8%) and 12 had cases that were pending (33.3%). Participants who were working at least a few hours a week worked in a variety of industries: food services (*n* = 6, 16.2%), building management (*n* = 5, 13.5%), construction (*n* = 4, 10.8%), peer specialists (*n* = 3, 8.1%), medical (*n* = 2, 5.4%), truck driving (*n* = 2, 5.4%), student (*n* = 1, 2.7%), and other (i.e., factory, investments, and “entry-level;” *n* = 3, 8.1%). Eleven participants were not working at the time of the interviews.

Most participants reported having several mental illness and medical diagnoses. Table 1 outlines participant self-reported mental disorder diagnoses, other major medical problems, and substance use disorders. The most commonly reported diagnosis was Bipolar I (*n* = 13, 35.1%). Just about three quarters of participants had at least one major medical comorbidity (*n* = 27, 73.0%). Over half of participants had a co-occurring substance use disorder (*n* = 28, 75.7%).

**Table 1:** Mental health and medical diagnoses.

	***n***	**%**
Mental disorder diagnosis*		
Anxiety-related disorder	6	16.2
Major depressive disorder	12	32.4
Bipolar I	13	35.1
Schizophrenia or schizoaffective disorder	10	27.0
Post-traumatic stress disorder	6	16.2
Obsessive compulsive disorder	2	5.4
Oppositional defiant disorder or intermittent explosive disorder	2	5.4
Personality disorder	2	5.4
Has substance use disorder diagnosis	28	75.7
Has major medical co-morbidity	27	73.0
	***M***	***SD***
Number of medical conditions (range 0–20)	3.4	4.2

*Participants noted all applicable diagnoses, so percentages do not equal 100%.

Participants were arrested prior to the age of 18, on average, 3.6 times (*SD* = 9.3) with a range of zero juvenile arrests to a high of 25. The number of adult arrests ranged considerably from one to 150 (*M* = 24.4, *SD* = 33.5).

### Participant Treatment Experiences

All participants in this study utilized health or mental health services during their incarceration. These prison health experiences were shaped by interactions with correctional and healthcare staff. These interactions contribute to the ability to access and use services, whether a person's medical and mental health needs are taken seriously, and the quality of care they receive. We first discuss participants' experience with staff and then present a focused discussion on their treatment. In this study, participants discussed experiences with various staff including medical and mental health staff, substance use treatment providers, and correctional officers, all of whom play a role in accessing and engaging in treatment. Prominent treatment-related experiences included perceived access and quality of mental health, medical, and substance use treatments obtained during prison as well as participant's willingness to engage in services. Each code is detailed below with direct quotes from participants to illustrate the meaning of codes.

### Interactions With Staff

Staff interactions included perceptions of staff believing and dismissing medical concerns as well as rapport building with both treatment staff and other staff, like correctional officers. Participant perceptions of staff attitudes, ideas, communication, and behaviors they exhibited when talking to or interacting with people incarcerated were central to their treatment experiences. Participants did report variations in experiences with staff around relationship quality and the presence or absence of rapport. Staff were described as both “pretty nice,” “pretty good,” “pretty professional,” and “they're not ornery. They treat you like a human” and described as “very rude,” “racist,” “good cop and bad cop,” and “inconsistent.” The broad consensus was that most medical staff and officers were just doing their jobs, but that there were a select few who also thought their jobs included making everyone miserable.

Across interviews, participants identified staff communication as displays of respect or disrespect. Participants identified staff as rude or unprofessional based on the way staff talked to them: “Some were respectful. They would call you ‘gentlemen.' Some…would just be arrogant and disrespectful and bring their problems from home to their job…it is a stressful environment no matter how you look at it,” (*Roger*^2^). Within these experiences, participants identified the salient feelings of being de-humanized. For example, “they just treat you like crap every time you went up there to medical anyway, so it was like I just avoided it” (*Billy*) and “you're treated sometimes like you're a piece of junk. You're nobody,” (*Samuel*). Participants perceived, “The COs (i.e., correctional officers)…you have some that do care…but the majority of ‘em it's just like they turn their heads,” (*Lenny*). These de-humanizing interactions occurred in an already oppressive system, amplifying their impact. *Glenn* recognized the macro context where these interactions took place: “…all the ethics and the rules that you have and the structure, it's all gone. When you're a CO's property, when you're their property, that's it. And the mentally ill are getting treated very poorly…in prisons.” Participants recognized the stressful work environment and challenges staff faced, yet they also recognized the choice that staff have to be humane in their interactions:

I know you got a job to do but talk to me. Don't talk down to me. Talk to me like, you know, we're here…you got a job and I'm in jail but I'm still a man…talk to me as such and respect me as such. And that's where you can make change. (*Gary*).

#### Staff Disbelief and Dismissal

This basic disrespect is also related to two problematic types of interactions that often occurred during interactions with prison healthcare providers: not being believed and not getting problems addressed. Because everyone in the prison has been convicted of a crime, participants felt the default response from providers was disbelief about their medical and mental health complaints. Providers defaulted to the belief that individuals would fake ailments to get away from their housing unit or to get attention. While participants stated that some people may do this, they felt it did not warrant everyone to be treated this way. Participants perceived some staff to not care about their concerns (e.g., “they don't give two damns about you,” *Chris*) or to not take them seriously (e.g., “They say, ‘Well you're not running a temperature so get out of here,” *Lenny*). Participants identified treatment experiences that ranged from feeling brushed off or ignored to experiences that resulted in medical neglect. One participant informed the nurse that she was about to give him too much insulin; she suggested he was not aware of the proper dose. The mistake was caught quickly so the nurse could intervene to decrease the participant's sugar levels before long term impacts occurred.

Some participants expressed concern that rationing of care through dismissing them or being short-staffed was rooted in the belief that participants' lives do not matter due to their status in society. *Randall* provided an example:

But they're contract employees, for one. And, two, they fall into the same regime of what the guards think. The guards get in their ears: "He's a murderer. He's a pedophile.” Whatever. “Don't treat 'em right. Don't – throw the Hippocratic Oath to the side. Don't worry about him.”

Participants also reported that if an individual was perceived to be faking an illness or pushed back too forcefully on the doctor's recommendation they could receive punishment, such as being confined to their cell or sent to administrative segregation.

Disbelief was especially prominent with mental health concerns. *Randall* reported an encounter with custody staff who were transporting the participant to a mental health treatment wing:

…I remember two guards that came in to cuff me to take me over there (mental health unit) and the sergeant come to the door, said ‘Where you taking him?' ‘He's going to the < mental health unit>.' And the guard was like, ‘What? There ain't nothing wrong with him.'…I remember thinking to myself, ‘You have no idea.'

Staff's disbelief in people's concerns can have serious short- and long-term consequences to both health outcomes and future treatment engagement. *Joseph* detailed an encounter with mental health staff during one of his incarcerations. He initially had a challenging time seeking help but eventually requested it because his symptoms became hard to manage on his own. He explained:

All that happened is I saw a therapist who they immediately changed my diagnosis from depression to like drug induced dystonia type of thing…And I was just totally thrown back by the fact that like I said it was so humbling to have to go in and admit that maybe I have a problem. I'm not even sure if I do. I go from I'm not sure whether or not I have a problem and having to be honest and kind of work through that to I'm having to argue. They're telling me you don't have a problem. There's nothing wrong with you.

In this specific situation with *Joseph*, the prison staff missed an opportunity to intervene. Unmanaged depressive symptoms, especially in stressful prison environments, can quickly escalate. Once released, events like this could also discourage people from help-seeking with community providers.

#### Staff Rapport Building

On the one hand, participants felt dismissed and disrespected by some staff, but on the other, participants also found the opportunity to build rapport with staff. Participants perceived an ability to build rapport with officers when officers got to know them: “…for the most part, you can get along pretty good with the guards, especially if they're in your wing and stuff, and they get to know you,” (*Billy*). Perceiving people living in prison as people rather than their charge or as a number humanizes them and shapes staff engagement.

Participant perspectives of staff did differ by their experiences within different housing units and prisons. In particular, participants found staff on specialized units, like a mental health wing or treatment unit, more available for rapport building. They perceived that staff wanted to work there and wanted to have a better understanding of mental health or medical issues. They also perceived the demeanor of officers to be more respectful and professional at prisons with higher security (e.g., maximum security facilities). Alternatively, participants perceived officers at camps that primarily housed people with sex offense charges or minimum-security facilities as “jerks” and “rude.” This same pattern did not hold across other staff like health and mental health professionals.

### Treatment Experience

Staff interactions are intricately intertwined with the treatment experiences. This content helps define the environment that people are help-seeking within. This next section focuses more specifically on the uses of treatment for mental health and medical conditions. Participants shared varying experiences in accessing medical services, mental health care and substance use treatments. Participant perceptions of quality and effectiveness of treatments varied across and within interviews. Participants reported a range of treatment experiences from groups and classes for managing substance use, self-help groups with peers, monthly meetings with drug counselors, and meetings with psychiatry and social work to medication only or no treatment at all. Similarly, perceptions of the quality of mental health and substance use treatment varied from participants perceiving they “just med you to death” to “my counselor was on top of everything.” In this section, participants' experience with treatment engagement including concerns they had about treatment use, decisions around use of treatment, and how they navigated the need for treatment in light of concerns is also detailed.

#### Variability in Access to Services

Access to services was variable across the sample and within individual interviews depending on which prison participants discussed. Overall, participants did not perceive care in prison to be patient-centered or preventative, although some participants did find services to be “all right” and perceived that care “…might not be great. Might not be exactly what you want but you can get a reasonable amount of care.” Participants also described their healthcare as “cookie-cutter,” “minimal,” and that staff are “going through the motions.”

The time from request of services to receipt of services for acute health issues (e.g., headaches, panic attack, sore throat) varied from within 24 h to several months. In one state, some prisons had clinics that would triage requests quickly while other participants reported waiting several weeks to several months to see a medical or mental health professional once a request was made. *Chris* reported, “…when you put your sick slip in, you might see a doctor two or three months down the road cause it's so many people. They don't have time to come and see you.”

Some participants reported being treatment connected prior to prison and in between incarcerations while others received treatment only while in prison. Sharing records between community agencies and the prison appeared to be a challenge from the perspective of participants. This lack of information sharing resulted in some participants going without the medication they were on in the community while others reported little disruption in care when medication was the only form of treatment.

Participants discussed pre-existing issues like substance use problems and untreated trauma exposure that they had prior to prison. Access to treatment for substance use during prison was available for some participants who had drug-related charges; however, some participants described that they were unable to access substance use treatment because it was not ordered by their sentencing judge. Participants reported they were able to access psychiatry services for their trauma exposure that occurred prior to prison which included receiving a formal diagnosis of post-traumatic stress disorder (PTSD), medication, and a monthly session with a mental health provider. One participant described being diagnosed with bipolar disorder and schizophrenia while he was in prison. He perceived that the experience of being incarcerated brought on these conditions (i.e., “…due to just all the BS you had to go through and see, and it kinda messes with people here,” *Lenny*).

In most cases, correctional officers are gatekeepers to care. They can control access to medical slips, how quickly requests reach the clinics, and when to arrange for transport to the clinics. In fact, participants attributed the length of time between request for services and receipt of those services to the officers' behaviors. *Chris* identified several delays that resulted in people lacking confidence that their requests for care were received: “…you gotta fill out the sick slip and give it to the guard maybe and maybe he'll put it in the sick box, and they get it later or whenever they decide to pick it up.” Participants uniformly reported that it was incumbent on them to make requests for acute health needs and that unless there was a clear emergency (e.g., heart attack, suicide attempt), officers or other staff were reluctant to initiate health services on their behalf. *Wes* reported that he risked getting disciplined by disrupting the food line in order to bring attention to his need for medical care. He made a request for medical services a week prior for a spider bite but had yet to see a medical provider. During this time, the bite became infected, and he was in constant pain. He reported:

…I put in my < medical request>, and five days later on my way to chow…I sit down right in the middle of the walk, guard walked over there and nudged me with his boot…'What's your problem?' ‘I wanna speak to a white shirt.'…before the white shirt could get there another officer got down there and he got down at my level…'what's going on, bro?'…I'm like, ‘Bro, it hurts to walk, like literally'…He said, ‘We're gonna go down to the medical.'

Despite initially going through the proper channels, this participant had to resort to disruptive measures, by sitting in the middle of the walkway and refusing to move, to demonstrate to officers that his condition required medical care.

#### Variability in Quality

Participants reported that health and mental health needs were greater than capacity, so encounters with doctors and nurses often felt rushed. Correspondingly, the first line of treatment for most ailments included pain relievers, like aspirin. Participants perceived that medical staff were unlikely to prescribe costly medications or screening tests until a person made multiple visits to the infirmary. This resulted in individuals having to manage pain on their own. Participants also experienced extensive waits for specialized care. *Joseph* suggested, “…you really have to be kind of sick or in bad shape to get any kind of a test done. Like they're not going to do an MRI unless you can't walk.” For medical conditions, participants experienced limited options for care, with medical staff mostly relying on pharmacology for treatment. These experiences deteriorated trust in providers and deterred people from utilizing services. *Randall* provided an example of this: “My back's hurting. ‘Yeah, okay. Here's three Motrin. Go ahead. We treated you.' So most times I wouldn't even bother going.” Ineffective strategies were contrasted with the care people could access in the community. *Jerome* provided an example:

You know, in prison, it's just minimal. Um, it's what they can do because I think they're really hampered by, you know, security reasons, and stuff like that. But, you know, in the community, it's a lot more focused and a lot more intense. You know, in prison, it's just kind of like, “Well, okay. You feel suicidal. We'll let you sit in a cell for a week, then we'll pull you out and see how you feel then.”

Despite these negative experiences, some participants found the care in prison to be helpful to understand their mental illness and identify the correct diagnosis. *Lenny* reported that the psychiatrist in the prison was patient and educated him about his new diagnosis: “He just basically told me all the symptoms about it and showed me paperwork. We went down the list, and he showed me the effects and how most people get it, and just showed me stories of other people… it was genuine.”

Chronic illnesses were addressed with more regularity and often identified upon intake, but some participants reported gaps in services and medications during intake processes or transfers between prisons. Others reported cumbersome processes to get care for chronic conditions. *Billy* reported that he had been in and out of prison in the same state several times and he had been treated for chronic obstructive pulmonary disease (COPD) several times before. However, upon re-entering prison, he was required to go through a new diagnostic process which delayed his care. He described:

…they knew that I had COPD, I still had to go through the process of going through that diagnostics…you gotta put in a request to see the doctor…When I finally got in to see the doctor, and then they saw my records, which was 30 days later, by the time I go there, I'd already declared an emergency probably four or five times.

Participants also reported being switched from routine psychiatric medications or medical treatments because they were not on the state's formulary or perceived to be too costly. Sometimes the change in medication resulted in uncontrolled symptoms of an illness that had been managed in the community, as was the case with *Billy's* chronic COPD.

Even if participants were pleased with their treatment, some still reported barriers to receiving their medications and seeing a doctor. Lines to receive daily medical and mental health medications were often exceedingly long and time consuming. Waiting in these lines could interfere with work duties, which caused some participants to either quit their jobs or quit taking medications so they could keep their jobs. Participants reported similar waits to see the providers. *Sylvia* recalled, “Long waits…some days I might have been trying to see the doctor, maybe the whole three days and the fourth day, I was like, okay, I'm going to skip lunch to see the doctor.”

Participants also reported concerns about the quality of care from providers working in the prison system. There was the perception that the good providers do not stick around for long due to the working conditions. Participants also perceived that people who do continue to work in the prisons do so because they do not have a medical license or have been reprimanded by their professions, resulting in them not having a choice about where to work. The perception of some participants was that prison providers are on the bottom tier of their profession. For example, *Samuel* reported, “I didn't feel like they were professional doctors…the nurses don't even really seem like they really got their RN degrees.” Again, participants contrasted community care with their experience in prison: “…one of them ruined what was called a buckle…from a true dentist on the streets, he goes ‘Man, who worked on you?” (*Robbie*). Regardless of whether these perceptions are true, these beliefs can create distrust in prison health services and providers.

#### Treatment Concerns and Decision to Engage

Participants reported many concerns about receiving treatment, especially for psychiatric problems, while living in the prison environment. These concerns shaped their decisions to engage in treatment when given the option. One prominent concern identified was that certain treatments could increase personal safety risks in the prison environment. This concern centered around the sedative effects of psychotropic medications like antipsychotics. *Gary* summarized his concerns:

I'm just afraid of it. They tried to give me Seroquel and I took it a little while, but you wake up in the morning, you're groggy…I had to stop taking it because I wasn't…feeling right…especially when you're locked up, you want to have all your faculties.

The safety concern was intertwined with concerns about being stigmatized, both self-stigma and stigma from staff and other people incarcerated. *Lenny* talked about decision-making surrounding taking medications while he was in custody:

Well, they talked to me about it (taking medication), but I used to see a lot of the guys how they were, and they called 'em *wobble heads*, and I didn't wanna be like that, especially in prison, you know, off guard and stuff like that.

*Juan* further identified the stigma, suggesting “if you take psych meds you'll be labeled as a wobble head.” Participants perceived this stigma stemming mainly from custody staff and other people incarcerated but not from health or mental health staff.

Treatment concerns also stemmed from the lack of privacy within the prison setting. Privacy is mostly unachievable which often deterred people from engaging in treatment, primarily mental health treatment. In some prisons, participants described mental health providers conducting sessions or check-ins in or near a person's cell, which allowed other staff and people incarcerated to observe the interaction. *Billy* identified the process for medication distribution as both public and uncomfortable, eventually causing him to stop taking medication: “I got tired of waiting in line to take a pill every night because a line would be 75 guys, so you might stand outside in the cold or the rain for an hour waiting to get a pill.” Beyond the discomfort of waiting in line in the cold and rain, this also offers a public forum for other people incarcerated to view who was taking psychiatric medication.

Privacy was also a barrier for treatment engagement in group settings. *Robbie* described the dual role that staff played where they may hear therapeutic processing during a group session and then use that information to report incidents. The reporting of incidents could lead to a violation or some other sort of punishment, which potentially deterred people from being open and honest: “…community treatment (is) much better...you don't have officers and staff watching over you. Because if you say or do the wrong things, you get in trouble” (*Robbie*). The lack of privacy is also problematic for people who may be struggling to manage or hide their illness. Stigma of mental illnesses is a constant presence in prison; participants described hiding their symptoms, refusing treatment, and not asking for help as strategies for keeping their mental illnesses invisible to others. *Sylvia* struggled to keep psychotic symptoms hidden:

It was hard because I'm in a cell with somebody and sometimes…just trying to get past like the voices and stuff…because I'm hearing them, I'm thinking I'm seeing things, and where do you go?...So, sometimes I talk back a little. Because I was really embarrassed about it and I cried a lot because I was so sick of it.

Finally, participant treatment concerns were also clustered around the cost of treatment and services. In some of the prisons, contact with treatment providers is a billable service to the incarcerated person. Refusing or opting out of care occurred for some because people did not have money for the service or because they did not want to be charged for the service. *Mitchell* noted, “…a few times I refused it (medical care) because I had no money.” Few participants described the actual cost to them, but *Kimmie* did recall:

You don't want to go see the nurse. No. If you see the nurse that's another $25 going on to your account…They charged the account if I have to get medicines, things like that. Sometimes they don't even get you (to take the medicine).

Given the overrepresentation of people living in poverty in the prison system, the cost of care could have widespread impacts on use of needed treatments and may contribute to the health impacts of incarceration.

### Navigating the Healthcare System

Participants reported several strategies to counteract their concerns about access, quality, and treatment. Participants recognized that problems with the access and quality of medical and psychiatric care were not necessarily due to the individual staff. The carceral system itself, as one participant noted, was the problem (e.g., “prison system not the medical system”) because the prison structure is set up to make medical intervention ineffective, through a combination of indifference, inaccessibility, and inertia. In the face of these systemic challenges, the strategy employed most often by participants was to forego medical and psychiatric care. Participants reported that they learned not to make requests for minor health needs as the ailment was likely to pass before they saw a nurse or a doctor. As mentioned above, some participants reported that they were charged fees for care which caused them to reconsider requests for treatment or refuse care when it was offered. Finally, concerns about being identified as someone with a mental illness and the accompanying stigma contributed to not seeking psychiatric or behavior health treatment.

For those determined to get their medical needs met, one strategy used was to “play the long game” by showing up repeatedly in medical and asking for treatment. Participants shared that they would not be deterred to pursue care by the lack of responsivity among the medical staff. Another strategy was to not seek care and let their condition go until the medical issue became an emergency. *Billy* provided an example:

I kept telling them that this really isn't gonna take care of the COPD stuff. They're so busy there, they don't care. I don't know how many times I declared an emergency there 'cause I couldn't breathe.

Emergency medical issues were prioritized and treated immediately. Some participants felt that this was the only way to secure care.

## Discussion

Health and mental health services have the potential to buffer the stressors and risks inherent in prisons for people with mental illnesses. Perceptions from participants suggest both individual- and systems-level opportunities for intervention to better support people with mental illnesses while they are in prison. Participants experienced dehumanization and stigma in attempts to receive care and perceived treatments to be inadequate in some cases. Access to health care was marked by cumbersome and time-consuming procedures required for service use requests and inadequate staffing. Participants reported mixed experiences with medical and mental health staff ranging from experiencing kindness to feeling staff did not believe them. Participants perceived some correctional officers as exhibiting professionalism while others enacted stigma and created additional stressors. Although not explored in this study, future research is needed to explore whether these experiences vary by gender or racial groups.

System-level barriers stemming from controlling the prison population resulted in de-humanizing and stigmatizing behaviors from staff and seeped into the prison health care settings, shaping both access and quality of medical and psychiatric care. Participant narratives suggested cost concerns and containment in a rationing of healthcare services by frontline workers and medical staff that ultimately influenced their care. This may be rooted in state budget concerns yet also may be attributable to for-profit medical services provided by private, contracted companies who may be attempting to minimize the costs of care (19).

In deciding how to ration services, prison healthcare workers operate as street-level bureaucrats because they have elevated levels of discretion and autonomy around the interpretation of prison policy and distribution of health services (33, 34). With the growing narrative that people with mental illnesses should be diverted from the prison system, it is possible that people's mental health diagnoses played a role in shaping their worthiness of receiving treatment (35, 36). Participants in the study were generally pleased with the mental health services they received through the prison, but less so with the physical health services. Further research could explore to what extent satisfaction with physical health services varies among those with and without mental health diagnoses and if there are differences in experiences across other salient factors (e.g., by gender or racial or ethnic groups).

A chief challenge faced by people with mental illnesses within the prison system was tension between the benefits and dangers of disclosing their mental illnesses. For example, participants perceived that having a mental health diagnosis documented upon admission or being on a specialized mental health unit improved how people were treated by correctional staff. However, the stigma of having a mental illness caused some people to forego medication and other types of mental health treatment, a finding that is echoed in the existing literature (37). People were particularly concerned that the impacts of psychotropic medications might leave them groggy and make them a target for violence or someone who could be taken advantage of because of their impaired ability to defend themselves.

Because of barriers such as stigma, medication side effects, costs of treatment, and general difficulty accessing regular care, some people responded by disengaging in treatment. In a similar dynamic to what happens when people disengage from healthcare systems outside prison, the result can be an over-reliance on costly emergency services. Beyond the fiscal implications, this may result in long-term health consequences that continue to impact people far beyond their stays in prison.

### Limitations

The aim of this study was not to recruit a representative sample of people with mental illnesses who have been incarcerated. Rather, participants were purposively sampled in order to gain understanding of the experiences of people using mental health and medical services and interacting with staff within prison. As such, findings from this study may not capture the broader experience of using prison treatment and support services. In addition, participants were asked to recall and think back on their experience in prison. It is possible that they were not able to recall all the details accurately. However, researchers did work carefully to ask participants questions in multiple ways to explore their experiences rather than requiring detailed knowledge of specific events. There was also variation in the experiences that people had with health and mental health care services and staff in prison. Reasons for variation including micro-level factors like race and gender were not explored so conclusions cannot be drawn about any potential causes for variation.

### Implications

This is one of few studies to explore how people with mental illnesses experience health care services and their interactions with staff in the prison environment. Understanding these dynamics leads to both individual- and system-based opportunities to help shape policies and future practice. As shown in this study and research from Pew (19), the accessibility, amount, and quality of health and mental health services is not consistent across prisons. Best practices and proper oversight are needed to ensure that prisons are not simply meeting the bare minimum standards but are promoting patient-centered and effective medical and mental health care. Just as community health and mental health providers and other providers of institutional care (e.g., nursing homes) are expected to maintain standards of care, so, too, should prison health care systems. National organizations in the United States like the National Commission on Correctional Health Care (NCCHC) have developed standards for jails and prisons on service delivery, quality improvement, patient safety, and treatments. Oversight and accreditation are currently voluntary but organizations like NCCHC have tools in place for prisons that want to be proactive in improving their health care systems.

Participants in this study faced barriers to accessing care for their mental and physical health needs. Addressing needs in prison can help people successfully re-integrate back into the community and decreases recidivism risks (5, 16). It is well worth the financial capital and time investment to improve the state of prison health services. Participants in this study perceived limited staff and rationing of services as contributing to poor care. Improving the prison health system by adequate financing and addressing concerns of privacy and stigma will also help increase engagement in these services. Participants in this study reported lengthy delays and a lack of trust in the health care services offered at the prison. Participants were concerned about their safety due to side effects of medications. Not accessing needed mental health services, for example, increases the risk of exacerbated psychological distress and suicide while incarcerated and upon exit from prison (13). The identified concerns from participants creates a slippery slope of medical and mental health needs not being addressed and further cultivating a culture that avoids accessing these vital services.

Participants in this study reported long wait times to see providers and “cookie cutter” approaches to care. Preventative, patient-centered services and reduced wait times to see providers may head off emergency services usage and prevent longer term health concerns that impact people who are incarcerated (7). Preventative care can benefit people incarcerated yet without proper support to prison health care workers, this may unintentionally create more strain on the system resulting in longer waits and more barriers to care. Currently, participants in this study perceive care is easiest to access in emergency situations. Emergency intervention and the treatment of longer-term medical issues caused by a lack of early intervention is costly. The use of preventative medicine would require prisons to shift costs from other services and into the healthcare system, but this shift has the potential to save health care costs in the long term and improve the quality of services offered to people incarcerated (38).

Just like in the community, mental illness stigma creates barriers to mental health care in prison. Participants in this study perceived stigma from both staff and people incarcerated. Prison staff may benefit from training to increase knowledge about mental illness and reduce stigma. A recent study on crisis intervention team (CIT) implementation in prisons found CIT reduces mental illness stigma among correctional officers (39). Additional information about mental illness could also help people in leadership positions better understand the urgency and necessity of mental health care. Changing policy and procedure to increase privacy can increase the number of people willing to engage in treatment. Although these practices may increase a person's willingness to access treatment, it is essential to ensure that enough providers and services are available for the people who need them.

At the individual level, communication between healthcare workers, staff, and people who are incarcerated could better establish trust and rapport. Since interactions with staff shaped accessing health resources from participant perspectives in this study, it is important for staff to recognize the implications of their interactions. Health care personnel may also benefit from training that addresses use of language and stigma about people incarcerated. Just like correctional officers, these interactions with health care staff may create barriers for people accessing needed care while in prison and could contribute to the health and mental health disparities among people incarcerated during and after their incarceration (7, 13, 21).

Individuals with mental illnesses are overrepresented in prisons, necessitating additional health care services within prison systems. Ensuring they are accessible, adequate, and reliable will not only improve health outcomes during incarceration but may also reduce the reliance on the criminal-legal system to address inadequacies in community systems contributing to mass incarceration.

## Data Availability Statement

The original contributions presented in the study are included in the article/supplementary materials, further inquiries can be directed to the corresponding author.

## Ethics Statement

The studies involving human participants were reviewed and approved by University of Missouri Institutional Review Board, New York University Institutional Review Board, and West Chester University Institutional Review Board. The patients/participants provided their written informed consent to participate in this study.

## Author Contributions

KC project conceptualization, data collection, data analysis, wrote abstract, method, results, implications. SB data collection, data analysis, wrote background and assisted with method and results. CB data collection, wrote discussion section and reviewed whole paper. AB data analysis, literature search, assisted with writing background and discussion. PP data analysis, literature search, assisted with writing implications. All authors contributed to the article and approved the submitted version.

## Funding

This work was partially funded through the University of Missouri Research Board.

## Conflict of Interest

The authors declare that the research was conducted in the absence of any commercial or financial relationships that could be construed as a potential conflict of interest.

## Publisher's Note

All claims expressed in this article are solely those of the authors and do not necessarily represent those of their affiliated organizations, or those of the publisher, the editors and the reviewers. Any product that may be evaluated in this article, or claim that may be made by its manufacturer, is not guaranteed or endorsed by the publisher.

## Footnotes

^1^These percentages are based on the participants who identified their race and ethnicity. Six participants did not complete a survey so they were omitted from the calculations.

^2^Researchers created pseudonyms for study participants.
